# The Complex Role of Mast Cells in Head and Neck Squamous Cell Carcinoma: A Systematic Review

**DOI:** 10.3390/medicina60071173

**Published:** 2024-07-19

**Authors:** Sofia-Eleni Tzorakoleftheraki, Triantafyllia Koletsa

**Affiliations:** Department of Pathology, School of Medicine, Aristotle University of Thessaloniki, 54124 Thessaloniki, Greece; sofialenatzo@gmail.com

**Keywords:** tumor microenvironment, genomic alterations in HNSCC, prognostic signatures, targeted therapy, oral squamous cell carcinoma

## Abstract

*Background and Objectives*: Head and neck squamous cell carcinoma (HNSCC) is a heterogeneous malignancy influenced by various genetic and environmental factors. Mast cells (MCs), typically associated with allergic responses, have recently emerged as key regulators of the HNSCC tumor microenvironment (TME). This systematic review explores the role of MCs in HNSCC pathogenesis and their potential as prognostic markers and therapeutic targets. *Materials and Methods*: A systematic search was conducted in the PubMed, Scopus and ClinicalTrials.gov databases until 31 December 2023, using “Mast cells” AND “Head and neck squamous cell carcinoma” as search terms. Studies in English which reported on MCs and HNSCC were included. Screening, data extraction and analysis followed PRISMA guidelines. No new experiments were conducted. *Results*: Out of 201 articles, 52 studies met the inclusion criteria, 43 of which were published between 2020 and 2023. A total of 28821 HNSCC and 9570 non-cancerous tissue samples had been examined. MC density and activation varied among normal tissues and HNSCC. Genetic alterations associated with MCs were identified, with specific gene expressions correlating with prognosis. Prognostic gene signatures associated with MC density were established. *Conclusions*: MCs have arisen as multifaceted TME modulators, impacting various aspects of HNSCC development and progression. Possible site-specific or HPV-related differences in MC density and activation should be further elucidated. Despite conflicting findings on their prognostic role, MCs represent promising targets for novel therapeutic strategies, necessitating further research and clinical validation for personalized HNSCC treatment.

## 1. Introduction

Head and neck squamous cell carcinoma (HNSCC) is the most common malignancy that develops in the mucosa of the oral cavity, pharynx and larynx [1]. It is a heterogeneous disease and smoking, alcohol consumption or previous exposure to oncogenic strains of the human papilloma virus (HPV) are well-documented risk factors [1,2]. HNSCC development follows a histologically multistep process from hyperplasia to invasive carcinoma. Various genomic changes lead to progression along this pathway, some of which are common and globally found in HNSCC, while others are restricted to one of the HPV-positive or HPV-negative categories [3]. The incidence of the disease is estimated to increase by 30% until 2030 [4], emphasizing the urgency of new prognosticators or treatment response predictors, or even predictors of the dynamics of a premalignant lesion.

In this concept, one of the strategies is to investigate both tumor cells and the tumor microenvironment (TME). The TME is a complex ecosystem comprising immune cells, which are active participants in all stages of cancer evolution. The TME represents the interactions between the neoplasm and the host’s defense in the “immune combat scenarios”. Mast cells (MCs) are the usual participants of this ecosystem [5]. Their size ranges from 6 to 12 μm, and their cytoplasm is filled with basophilic granules, which store a number of mediators comprising amines, proteoglycans and proteases, [6] as well as reacting substances of anaphylaxis and eosinophil chemotactic factors [7]. There are two main MC subtypes, namely mucosal and connective tissue MCs [8]. There are substantial differences between them, either in size [8] or in the content of their granules, possibly reflecting functional differences [9,10].

In regards to their functional abilities, MCs constitute a fundamental part of the innate immune response and interfere with other molecules throughout the adaptive immune response [11]. Thus, MCs are considered coordinators of immune responses. The main path that leads to MC activation is the IgE-FcεRI bond [12,13]. MCs are well known for their crucial role in immediate hypersensitivity (anaphylaxis) [10], in bacterial, viral, parasitic and fungal infections [14,15], as well as in autoimmune diseases and cancer [16,17,18]. As part of the TME, MCs seem to impact the prognosis of patients with malignancies [17]. MC recruitment and activation lead to extracellular matrix degradation through protease release and facilitate invasion and metastases [19]. Of note, MCs have been associated with smoking and alcohol in several organs [20,21], which are the main risk factors of HNSCC. In this context, MCs seem to have a crucial role in HNSCC carcinogenesis. In this systematic review, we aimed to identify original articles investigating MCs in HNSCC, in an effort to unravel their complex role and their potential as prognosticators.

## 2. Materials and Methods

### 2.1. Literature Search

A systematic search of the PubMed, Scopus and ClinicalTrials.gov databases was conducted until 31 December 2023, in order to identify relevant articles. The Medical Subject Headings (MeSH) and search terms used were “Mast cells” AND “Head and neck squamous cell carcinoma”. This systematic review was conducted in accordance with the Preferred Reporting Items for Systematic Reviews and Meta-Analyses (PRISMA) guidelines [22]. All research was conducted according to a protocol registered in the OSF database (registration number: https://osf.io/d8aqw/ accessed on 22 May 2024). The quality of the eligible studies was evaluated using the Risk of Bias In Non-randomized Studies—of Exposure (ROBINS-E) tool [23]. The risk of bias assessment was carried out by the authors and any disagreements were resolved by consensus. Pertinent data and the risk of bias are shown in Supplementary File S1.

This article does not contain any newly conducted experiments involving humans or animals; instead, it is based on previously published research. An ethics review is not required for this systematic review of published and non-identifiable data.

### 2.2. Study Selection and Data Extraction

All studies reporting MCs and HNSCC were included following the application of an English language filter. The search on the ClinicalTrials.gov database produced no results. Results from the other two databases were screened by the authors independently based on the predetermined criteria. Specifically, studies were considered eligible if they met the following inclusion criteria: (i) corresponding to original articles, (ii) providing information for both MCs and HNSCC, (iii) concerning primary HNSCC, and (iv) performed on human tissues. The exclusion criteria included: (i) reviews, (ii) case reports, (iii) comments or conference abstracts, (iv) animal or in vitro studies, and (v) metastatic or relapsed HNSCC.

**Figure 1:**
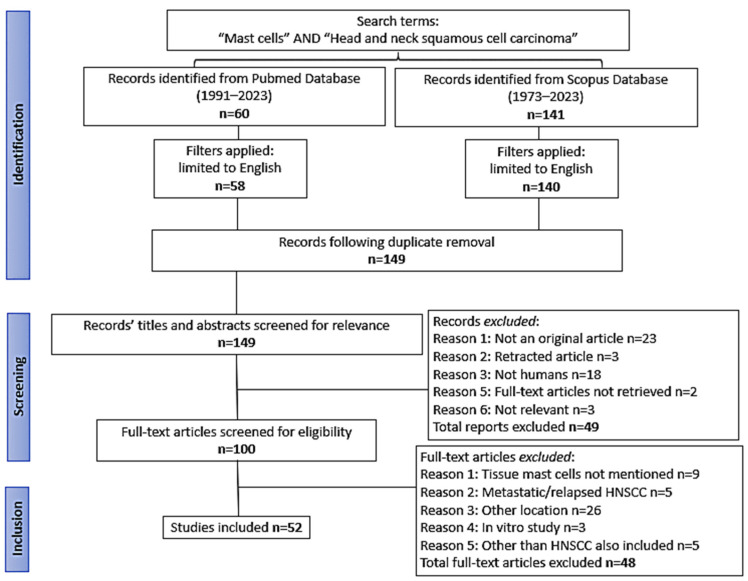
Flow chart of the study selection.

Each author evaluated the abstracts independently and created a list of papers that required detailed review. The lists were compared, and any discrepancies were worked out by consensus. Subsequently, the articles were read comprehensively and the following data were recorded in a predetermined form: name of the first author, publication year, study design, site of HNSCC, main result regarding MCs, MCs in cancerous vs. corresponding normal tissue, and MC association with specific genomic alteration and their prognostic impact. The screening method and results are illustrated in Figure 1.

## 3. Results

In this systematic review, the recent interest in the role of MCs in HNSCC is highlighted. Variations in MC density and activation were observed between normal and cancerous tissues, with specific genetic alterations and gene expressions correlating with prognosis. MCs are emerging as promising targets for novel therapeutic strategies, necessitating further research and clinical validation for personalized HNSCC treatment.

### 3.1. General Data of the Studies

A total of 201 articles were initially retrieved from the PubMed, Scopus and ClinicalTrials.gov databases, 198 of which were written in the English language. A total of 49 duplicate articles were removed and an additional 49 were excluded based on the title and abstract. After careful review of 100 full-text articles, 52 studies that met all criteria were finally included in this systematic review (Table 1). Of note, 43 of them were published between 2020 and 2023, although the first report on the issue was in 2000. A total of 28821 HNSCC along with 9570 non-tumor tissue samples were included in the eligible studies. Non-tumor tissues were characterized as normal (n = 9202), dysplastic/premalignant (n = 32) or non-neoplastic adjacent to tumor tissue (n = 336).

**Table 1:** Comprehensive analysis of publications reporting on HNSCC MC infiltration.

A/A	First Author, Year [Ref. No.]	HNSCC Anatomical Site	Method	Reported Gene Alteration or Signature	Main Result Regarding Mast Cells
1	Zhao C, 2022 [24]	HNSCC	MOL	*MRGBP*	High MRGBP was associated with MCs and adverse prognosis.
2	Zhang X, 2022 [25]	HNSCC	MOL/database repository	*CBX3*	CBX3 expression was negatively correlated with MCs.
3	Zhang S, 2022 [26]	HNSCC	MOL/database repository	Risk model established by the incorporation of 8 genes related to CD8^+^ T cell infiltration	High-risk group was characterized by a lower immune score and abundant aMCs and presented with adverse prognosis.
4	Han S, 2021 [27]	HNSCC	MOL/database repository	Risk model established by the incorporation of 191 genes related to SUVmax on ^18^F-FDG PET/CT	High-SUV group was characterized by a lower immune score and weak MC density and presented with adverse prognosis.
5	Li J, 2023 [28]	HNSCC	MOL/database repository	*HOXB9*	Low HOXB9 expression was significantly associated with an increase in the proportion of MCs.
6	Peng C, 2023 [29]	HNSCC	MOL/database repository	*GAPDH*	Elevated GAPDH expression hindered communication between pDC and MCs.
7	Sawatsubashi M, 2000 [30]	Larynx	IHC	ND	VEGF staining in squamous cell carcinomas was associated with MC count. Laryngeal cancer cells and MCs may control the angiogenic response by releasing VEGF.
8	Featherston T, 2017 [31]	Oral	IF	ND	Cathepsin G was localized to the tryptase+ phenotypic MCs within the peri-tumoral stroma.
9	Chang SR, 2023 [32]	HNSCC	MOL/database repository	Immune checkpoint-related genes	Decreased aMCs were common in tumors compared to normal tissues and found in advanced oral squamous cell carcinoma.
10	Wang P, 2023 [33]	HNSCC	MOL/database repository	*PGK1*	PGK1 was negatively correlated with the level of MC infiltration.
11	Hess AK, 2017 [34]	Oropharynx, hypopharynx	MOL	miRNA array and immune expression panel	There was no correlation between miR-146a/miR-155 expression and MC infiltration.
12	Rhee JK, 2023 [35]	Oral	MOL/database repository	CIBERSORTx gene expression profile which quantified the composition of 10 cell subtypes	MC density did not show obvious statistical differences between the tumor tissues and the normal surgical margins.
13	Barth PJ, 2004 [36]	Oral cavity, pharynx, larynx	IHC	ND	The number of tissue MCs was significantly increased in carcinomas compared to tumor-free mucosa.
14	Cosoroabă RM, 2022 [37]	HNSCC	IHC	ND	MC density was significantly higher in carcinoma compared to dysplastic lesions and control cases.
15	Jonsson EL, 2012 [38]	Oral, oropharynx, hypopharynx	IHC	ND	Pre- and post-RT tumors showed an abundance of MCs compared to the corresponding normal tissues.
16	Zhou D, 2021 [39]	HNSCC	MOL/database repository	Immune-related genes	HPV (+) tumors were richer in resting and aMCs and HPV (-) tumors showed higher rMCs and lower aMCs compared to normal tissues.
17	Liang B, 2020 [40]	HNSCC	MOL/database repository	A leukocyte gene signature matrix (LM22)	rMCs were significantly lower in advanced T stage. The proportion of aMCs in HNSCC tissues was significantly higher than in adjacent non-cancer tissues.
18	Liu Z, 2020 [41]	Oral	MOL/database repository	*FOXD2-AS1*	FOXD2-AS1 was involved in tumor progression via epithelial-to-mesenchymal transition and cell cycle regulation and was negatively associated with MCs.
19	Sun Y, 2022 [42]	Oral	MOL/database repository	*DDX59-AS1*	The expression of DDX59-AS1 was negatively correlated with MCs.
20	Han Y, 2021 [43]	Oral	MOL/database repository	*MFAP4*	MFAP4 gene expression showed positive correlation with MC infiltration. Higher MC infiltration correlated with better survival.
21	Chen C, 2022 [44]	Oral	MOL/database repository	*SFRP1*	SFRP1 displayed a positive performance in tumor immune infiltration, especially in MCs.
22	Sobocińska J, 2022 [45]	HNSCC	MOL/database repository	*ZNF418* and *ZNF540*	Patients with a higher expression of ZNF418 and ZNF540 genes displayed lower levels of MCs.
23	Li C, 2023 [46]	HNSCC	MOL/database repository	*MYL1*	MYL1 expression was positively correlated with MCs.
24	Jin Y, 2020 [47]	HNSCC	MOL/database repository	ND	rMCs were closely correlated with HNSCC progression.
25	Jin Y, 2023 [48]	HNSCC	MOL/database repository	Risk model established by the incorporation of circadian genes	High-risk group was characterized by abundant aMCs and was correlated with adverse prognosis, whereas abundant rMCs were associated with favorable overall survival.
26	Stasikowska-Kanicka O, 2017 [49]	Oral	IHC	ND	Oral squamous cell carcinoma with adverse prognosis exhibited significantly lower mean number of MCs.
27	Attramadal CG, 2016 [50]	Oral	MOL/database repository/IHC	c-KIT, SCF and genes encoding for MC tryptases	High-budding tumors had lower MC density and relapsed more frequently. High expression of c-KIT- and SCF-mRNA associated with better 5-year survival.
28	Almahmoudi R, 2018 [51]	Oral	IHC	ND	The extracellular MC-derived IL-17F at the tumor invasion front of oral tongue squamous cell carcinoma was associated with better disease-specific survival.
29	Ishikawa K, 2014 [52]	Oral	IHC	ND	MC density was associated with adverse prognosis and correlated significantly with IL-33 expression.
30	Yang C, 2022 [53]	HNSCC	MOL/database repository	Risk model established by the incorporation of 9 hypoxia-related lncRNAs	High-risk group was characterized by abundant activated and few rMCs and presented with adverse prognosis.
31	Chen Q, 2021 [54]	HNSCC	MOL/database repository	Risk model established by the incorporation of 6 fibrosis–hypoxia–glycolysis-related genes	High-risk group was characterized by abundant MCs and presented with adverse prognosis.
32	Li Q, 2022 [55]	HNSCC	MOL/database repository	Risk model established by the incorporation of 35 differentially expressed immune-related (Deir) lncRNAs	High-risk group was correlated with high aMCs infiltration and low infiltration of rMCs and presented with adverse prognosis.
33	Chen F, 2022 [56]	HNSCC	MOL/database repository	Risk model established by the incorporation of 4 aging-related genes	High-risk group had lower immune score than low-risk group, was characterized by abundant resting and few aMCs and presented with adverse prognosis.
34	Sun Q, 2021 [57]	HNSCC	MOL/database repository	Risk model established by the incorporation of 17 T-reg-related lncRNAs	High-risk group was characterized by abundant aMCs and presented with adverse prognosis.
35	Fan X, 2021 [58]	HNSCC	MOL/database repository	Risk model established by the incorporation of 17 ferroptosis-related genes (*PR-DE-FRGs*)	High-risk group was characterized by a lower immune score and weak MC density and presented with adverse prognosis.
36	Ding Z, 2021 [59]	HNSCC	MOL/database repository	Risk model established by the incorporation of 24 hypoxia-related genes	High-risk group was characterized by abundant activated and few rMCs and presented with adverse prognosis.
37	Fan X, 2023 [60]	HNSCC	MOL/database repository	Risk model established by the incorporation of 6 endoplasmic reticulum stress-related genes	High-risk group was characterized by abundant aMCs and presented with adverse prognosis.
38	Ding Y, 2023 [61]	HNSCC	MOL/database repository	Risk model established by the incorporation of 9 immune infiltration-related genes	High-risk group was characterized by increased aMC infiltration and presented with adverse prognosis.
39	Cai Z, 2022 [62]	HNSCC	MOL/database repository/FISH	Prognostic MC signature comprising 9 genes	High-risk group was characterized by low immune infiltration including MCs and presented with adverse prognosis.
40	Dong L, 2023 [63]	Oral	MOL/database repository	Risk model established by the incorporation of 8 CAF-related genes	High-risk group was characterized by high infiltration of aMCs and low infiltration of rMCs and presented with adverse prognosis.
41	Yuan Z, 2022 [64]	HNSCC	MOL/database repository	Risk model established by the incorporation of 22 fatty acid metabolism (FAM) genes	High-risk group was characterized by high infiltration of aMCs and presented with adverse prognosis.
42	Wang E, 2021 [65]	HNSCC	MOL/database repository	Risk model based on m6A/m5C/m1A-related long non-coding RNAs (lncRNAs)	High-risk group was correlated with high aMCs infiltration and presented with adverse prognosis.
43	Sun Q, 2023 [66]	HNSCC	MOL	Risk model established by the incorporation of 39 cuproptosis-related lncRNAs	High-risk group was characterized by lower MCs and presented with adverse prognosis.
44	Zhou S, 2022 [67]	Larynx	MOL	Risk model established by the incorporation of 12 microRNA pairs	High-risk group was characterized by higher aMCs proportion and presented with adverse prognosis.
45	Tao ZY, 2024 [68] *	Oral, pharynx	MOL/database repository	Risk model established by the incorporation of 4 neural-related genes	High-risk group was characterized by high infiltration of rMCs and weak infiltration of aMCs and presented with adverse prognosis.
46	Li YJ, 2022 [69]	HNSCC	MOL/database repository	Risk model established by the incorporation of 10 cuproptosis-related lncRNAs	High-risk group had lower immune score than low-risk group, was characterized by abundant activated and few rMCs and presented with adverse prognosis.
47	Wu L, 2023 [70]	Oral	MOL/database repository	CAF-related risk model based on TISCH2-single-cell RNA sequencing analysis	MCs were closely correlated with the CAF-based periodontal disease-related risk model.
48	Huang J, 2021 [71]	Oral, Larynx	MOL/database repository	*FOXD1*	The FOXD1 high-expression group was significantly associated with the proportion of aMCs and with adverse prognosis.
49	Cui L, 2020 [72]	HNSCC	MOL/database repository	*MTHFD2*	Increased MTHFD2 expression was positively correlated with MC activation.
50	Liu Y, 2021 [73]	Oral	MOL/database repository	*SQLE*	aMCs were negatively correlated with SQLE mRNA expression.
51	Chu M, 2022 [74]	HNSCC	MOL/database repository	*SLC2A3*	High SLC2A3 expression was associated with MC infiltration and adverse prognosis.
52	Lin YH, 2022 [75]	HNSCC	MOL/database repository	*FCGBP*	The FCGBP mRNA level positively correlated with MC infiltration rates.

Ref No: reference number; aMCs: activated mast cells; CAF: cancer-associated fibroblasts; HNSCC: head and neck squamous cell carcinoma; HPV: human papilloma virus; IF: immunofluorescence; IHC: immunohistochemistry; MCs: mast cells; MOL: molecular; ND: not determined; pDC: plasmacytoid dendritic cells; rMCs: resting mast cells; RT: radiotherapy; SCF: stem cell factor. * Electronic publication (Epub) in 2022.

Eight studies used immunohistochemical methods, along with mRNA investigation for c-KIT expression in one of them, another one performed immunofluorescence, whereas forty-three analyzed records from molecular databases. The majority of the studies (71.2%) included cases from more than one head and neck sites, while thirteen (25%) encompassed only oral carcinomas and two (3.8%) only laryngeal. In addition, MCs were characterized as activated or resting in 21 articles, whereas the remaining 31 referred to total MC infiltration, without further categorization.

### 3.2. Site-Specific MC Infiltration Among Normal and Malignant Tissues

The comparison of MC density among normal and malignant tissues of several HNSCC sites was conducted in seven studies during the past decade [32,35,36,37,38,39,40]. Two of them investigated oral squamous cell carcinoma (SCC),and the rest of them included variable HNSCC sites. In regards to oral SCC, activated MCs were either reduced in tumors [32] or did not present significant quantity differences compared to normal tissues [35]. The remaining studies concluded that MCs were more abundant in HNSCC, compared to corresponding non-neoplastic tissues, and they were either activated [40] or of unknown activation status [36,37,38]. One of these studies reported that HPV-positive tumors were higher in resting and activated MCs and HPV-negative tumors were higher in resting but lower in activated MCs, albeit the results were not statistically significant [39]. Only one study compared MC proportions among sites and showed that higher MC densities were a feature of lip SCC [37].

### 3.3. Gene Alterations Associated With MCs in HNSCC

In total, 16 of the studies described genetic alterations associated with mast cell density or activation in the HNSCC TME and were published over a period of four years (Table 2). All of them utilized data from databases. Five of the studies included only cases from oral SCC, while the rest were from different head and neck sites.

Genes overexpressed by neoplastic cells have been associated with MC density or their activation status. Overall, these corresponded to transcription factors or were involved in cell metabolic processes or cell senescence or participated in intracellular pathways. In addition, specific long-stranded non-coding RNAs (lncRNAs) seemed to have a role in MC recruitment and activation [41,42]. The majority of all these genomic alterations were characterized as adverse prognosticators for HNSCC, whereas the high expression of only four genes, namely the *MFAP*, *SFRP1*, *ZNF418* and *ZNF540* genes, was identified as a good prognosticator [43,44,45]. The overexpression of the genes implicated in aging and metabolic processes, as well as of lncRNAs, was associated with dismal prognosis. Interestingly, a high expression of the human myosin light chain 1 (MYL1) gene, a tumor-promoting gene, was correlated with tumor immune infiltration and MC infiltration in HNSCC [46]. The status of MCs, either activated or resting, was characterized only in 4 of the 16 selected articles. Pertinent data are summarized in Table 2.

### 3.4. Prognostic Role of MCs in HNSCC

Overall, the prognostic role of MCs in HNSCC was investigated in eight studies over the past decade [26,43,47,48,49,50,51,52]. Five of them included oral sites, while the remaining comprised several HNSCC sites. The latter concluded that MCs, either activated [26,48] or resting [47], were associated with poor prognosis. On the other hand, specifically for oral SCC, four publications correlated high MC density with better survival, either in reference to the entire tumor [43,49] or exclusively to the invasive margin [50,51]. Only one study linked elevated MC density in oral SCC with worse prognosis [52].

### 3.5. Prognostic Gene Signatures Associated With MC Density

Twenty-one HNSCC risk models [26,27,48,53,54,55,56,57,58,59,60,61,62,63,64,65,66,67,68,69,70] were established, mostly by the incorporation of genes relevant to specific cellular functions, and the patients were divided into high- and low-risk groups (Table 1). Apparently, high-risk patients were correlated with poor prognosis. MCs were examined either uncategorized or subcategorized into resting and activated. Four publications concluded that low total MC density characterized the high-risk group, as opposed to a single study that supported the opposite. Thirteen studies supported that activated MCs were more abundant in the high-risk group; however, resting MCs exhibited higher numbers in three additional studies.

## 4. Discussion

MCs play a crucial role in HNSCC by influencing the TME through angiogenesis, tissue remodeling and immunomodulation [62,76]. They secrete various mediators that affect both tumor and immune cells, promoting or inhibiting tumor growth [77,78,79]. MC density and activation vary by tissue and tumor site, indicating site-specific differences [80,81]. Genomic alterations, such as MAPK or PI3K/AKT/mTOR mutations, may influence MC recruitment and activity, though the mechanisms are complex and need further research [82,83]. Prognostic implications of MCs in HNSCC are mixed, with some studies linking higher MC density to poorer prognosis, while others suggest anti-tumor effects [43,48,51]. Therapeutically, targeting MCs with inhibitors or modulators could enhance treatment efficacy, especially with anti-PD-1/PD-L1 inhibitors [84,85,86]. However, MC plasticity and site-specific differences necessitate personalized approaches in HNSCC treatment [62,87].

### 4.1. MCs in TME

MCs have gained significant attention in the context of cancer research over the past decade, including in HNSCC, a fact that is reinforced by this systematic review. While primarily viewed as mediators of allergic responses, MCs have emerged as complex regulators of the TME, capable of influencing various aspects of cancer development and progression [76,88]. There is a vivid scientific interest to elucidate their interaction with neoplastic cells and other components of the TME in order to identify or even intervene in their pro-tumorigenic or anti-tumorigenic status [62,76]. It is well known that MCs, upon activation, can secrete either by degranulation or by “selective” release a range of immune mediators [77]. This secretion can have both local and systemic effects, influencing the behavior of various immune cells and even the tumor cells themselves [78].

The complex role of MCs in HNSCC, as part of the TME, has been increasingly recognized [62]. MCs possess the ability to interact with a variety of cells and to secrete molecules in the tumor stroma [89], leading to their involvement in angiogenesis, tissue remodeling, immunomodulation and inflammation, and hence, potential tumor progression [79]. MCs produce multiple angiogenic factors including proteases (chymase and tryptase), basic fibroblast growth factor (bFGF), IL-8, transforming growth factor (TGF-β), TNF-α and vascular endothelial growth factor (VEGF), which stimulate neovascularization [90], a crucial process for supplying nutrients to the tumor and facilitating tumor promotion [91]. Specifically for oral carcinomas, there is a hypothesis of a positive correlation between microvascular and MC density [92]. Νeovascularization is enhanced by heparin, which binds to bFGF and TGF-β [93], and histamine, which interacts with H1 and H2 receptors [94]. Protease secretion has an additional major effect on tissue remodeling, as it activates matrix metalloproteinases (MMPs), resulting in peritumoral extracellular matrix distortion and, subsequently, in tumor spread acceleration [79]. Histamine and tryptase operate on the tumor cells, as well, and stimulate their proliferation [94]. Moreover, MCs release chemokines and cytokines which interfere with the systemic immune host response by altering the initial function of immune cells inhabiting the TME, such as B- and T-lymphocytes. This immunomodulation might either enhance or suppress tumor growth [95].

### 4.2. MCs in Normal Tissues and HNSCC

Several tumors of variable primary sites deliver conflicting results when comparing MC density between neoplastic and matched non-neoplastic tissue [80,81]. In addition, activated MC density may differ from resting MC density between tumors and corresponding normal tissue [96]. Τherefore, there might be site-specific differences regarding the presence or activation of MCs in various human organs. Similarly, in this systematic review, there was an apparent elevation of MC quantity in HNSCC, in contrast to oral SCC. In terms of site-specific HNSCC, oral SCC showed contradictory results with either a reduced or a non-significant change in the number of MCs in malignancies. These findings do not align with the results of the remaining sites and necessitate additional research. Only one study reported that lip SCC exhibited higher MC rates compared to the remaining HNSCC sites [37], a fact that could be attributed to the dermal MC recruitment following chronic sun exposure [97,98]. Even though there are a few studies investigating the TME immune cells among HNSCC sites, they mostly focus on TILs [99,100] and lack references to MCs. Importantly, there are limited data regarding the association of MC recruitment and HNSCC risk factors. In this line, whether chronic stress significantly induces increased MC density, as recently observed for T-lymphocytes [101], remains unknown and requires further investigation.

### 4.3. Genomic Alterations and MC Recruitment

MC recruitment is a complex process involving several molecules and signaling pathways. Some of the key molecules involved include chemokines, adhesion molecules, stem cell factor, cytokines or complement proteins [83]. Mutations in HNSCC may influence the recruitment of various immune cells including MCs. For instance, MAPK-mutated HNSCC have been associated with a fully-inflamed TME, characterized by high T-cytotoxic recruitment and activity [102,103]. Several studies, as presented above, have suggested that certain mutations could impact the recruitment and activation of MCs in HNSCC [71,72,73,74,75]. However, the relationship between specific mutations and MC recruitment in HNSCC requires further investigation.

It is emphasized that several recent studies have employed entire tumor gene expression profiles and deconvolution algorithms like CIBERSORT, trying to investigate whether molecular alterations influence the immune infiltrates. In this context, a high density of activated MCs has been reported in several studies occupying risk models like those based on hypoxia-related, cuproptosis-related or other lncRNAs [53,57,65,69] as well as ferroptosis-, hypoxia- or fatty acid metabolism-related genes [58,59,64]. The variability in genetic alterations and gene expression profiles associated with MCs among different studies may underscore the need for standardized approaches.

In addition, mutations in genes related to the PI3K/AKT/mTOR pathway or HER pathways have been implicated in modulating the TME in HNSCC [104], which could potentially influence MC recruitment. Ding et al. described a higher MC density in HPV-positive HNSCC [59]. In HPV-positive oral and oropharyngeal carcinomas, molecular mechanisms that promote oncogenesis are different from HPV-negative tumors [82,105]; however, the MC role in HPV lesions is uncertain. Overall, while specific mutations may contribute to the recruitment of MCs in HNSCC, the exact mechanisms are complex and require further investigation, integrating genomic, transcriptomic and immunological analyses.

### 4.4. Prognostic Impact of MCs

In this systematic review, it is concluded that findings regarding MC prognostic impact can vary between different studies and methodologies. Some studies suggest that increased MC infiltrates in HNSCC tumors may be associated with a poorer prognosis [48,52], as MCs can promote tumor growth, angiogenesis and immunosuppression. Conversely, other studies propose that mast cells may exert anti-tumor effects by enhancing immune responses against the cancer cells [43,51]. Furthermore, in the context of the recently described effect of the immune microenvironment on radioresistance [106], investigating MC influence on the efficiency of radiotherapy may offer insights into their impact on tumor survival and post-radiotherapy recurrence.

Factors such as the location of MCs within the TME, their activation status and their interactions with other immune cells and tumor cells may all influence HNSCC prognosis [63]. Additionally, activated MCs can recruit effector T-cells and natural killer cells or can influence B-cell activity [63], which support their ability to shape the overall inflammatory status of the TME and subsequently to modulate their prognostic impact. Regarding their role as good prognosticators in oral carcinomas, this could be attributed to HPV-related tumors that represent a distinct molecular entity characterized by higher survival rates [107,108].

In all, MCs are versatile cells possessing different morphologies and functions, mostly dependent on their interactions with neoplastic cells and the microenvironment. Large-scale clinical studies with standardized methodologies are essential for elucidating the precise relationship between MC infiltration and clinical outcomes in HNSCC patients.

### 4.5. Therapeutic Implications of MCs in HNSCC

The emerging role of MCs as coordinators of innate and adaptive immunity in the TME [62,76], as well as their interactions with several immune cells, makes them an attractive target to develop novel therapeutic strategies. There are several recent clinical and preclinical studies addressing this issue [46]. Considering their roles in tumor evolution that were discussed previously, as well as the pertinent literature, the immunotherapeutic options encompass the reduction of their number in the TME, modulation of their activation and phenotype and alteration of secreted mediators.

MC number and function could be controlled through tyrosine kinase inhibitors, including c-kit and BTK inhibitors. Whether obatoclax, a pan-bcl-2 blocker with direct antitumor effects, succeeds in decreasing MCs in the TME as well by inducing apoptosis, as shown in several MC lines, remains to be clarified. MC function could be modulated by tryptase inhibitors, such as gabexate mesylate, nafamostat mesylate and tranilast [80] or H1, H2 CysLT1 or PGD2 receptor antagonists [109,110]. In this context, TLR-agonists may alter their phenotype and actionability, as well as other agents, like cromolyn sodium, which have been associated with immunotherapy response [85]. In this context, a potential strategy to increase the effectiveness of anti-PD-1/PDL-1 treatment could be the inhibition of MC function [76]. Elevated PDL-1 levels in MCs could induce T-cell immunosuppression and tumor progression [86]. Therefore, the combination of anti-PD-1/PD-L1 inhibitors with a TKI seems to be a more effective therapeutic approach [76,111]

Several efforts are ongoing in order to develop a tool for characterizing MCs and utilize it as a predictive marker for immunotherapy response in HNSCC patients [62]. However, we should first take into consideration MC plasticity, as well as site-specific MC density, given the fact that high MC density is associated with longer overall survival in patients with oral SCC [87] compared to other HNSCC, along with their profiling.

In all, there are several MC-oriented anti-tumor strategies in the preliminary stage. The efficacy of these approaches in combination with other anti-tumor regimens warrants further investigation. Site-specific predictive tools and therapeutic options based on MC phenotype and function are promising treatment modalities in terms of personalized therapy in HNSCC.

### 4.6. Strengths and Limitations

The strength of this systematic review is its extensive search strategy, which follows PRISMA guidelines and guarantees a strict selection process of pertinent studies. We were able to capture the changing state of this field’s research over the years. Furthermore, the incorporation of research employing various approaches for MC recognition and/or characterization facilitated a more thorough examination of the subject.

However, the relatively small number of studies that fit our inclusion criteria, the approach to HNSCC as a single entity and not as a heterogeneous disease, as well as MC versatility could be considered limitations of the current systematic review. In addition, the heterogeneity of the included studies, comprising differences in study design, sample size and assessment methodologies of MC density and activation, complicates direct comparisons and potentially leads to inconsistent findings. It is emphasized that most studies are observational, limiting the ability to establish causality between MC presence and HNSCC progression.

## 5. Conclusions

Understanding the role of MCs in HNSCC is still an active area of research and their precise contributions may vary depending on the specific context of the TME. The findings of this systematic review are characterized by clearly conflicting information, which are really a result of the plastic nature of MCs that are amazingly sensitive to the microenvironmental signals to which they suddenly react. Their prognostic impact is influenced by their activation, degranulation, localization and cell-to-cell interactions. In the latter situation, specific mutations and prognostic gene signatures seem to play a role. In addition, substantial differences in MC densities and their prognostic roles are found between oral SCC and other HNSCC. Targeting MCs or their mediators represents a potential therapeutic strategy for HNSCC. However, further research is needed to elucidate their biology and to identify specific profiles related to either pro-tumorigenic or anti-tumorigenic roles.

## Figures and Tables

**Table 2:** Genomic alterations associated with MC infiltration in HNSCC TME.

A/A	First Author, Year	HNSCC Anatomical Site	Genomic Alteration	Prognostic Impact of Genomic Alteration	Correlation with Mast Cells
High expression of transcription factors					
1	Huang, 2021 [71]	HNSCC	*FOXD1*	Adverse prognosis	Positive correlation with aMC infiltration
2	Zhao, 2022 [24]	HNSCC	*MRGBP*	Adverse prognosis	Positive correlation with MC infiltration
3	Sobocińska, 2022 [45]	HNSCC	*ZNF418* and *ZNF540*	Favorable prognosis	Negative correlation with MC infiltration
High expression of genes involved in cellular metabolic processes					
4	Cui, 2020 [72]	HNSCC	*MTHFD2*	Adverse prognosis	Positive correlation with aMC infiltration
5	Liu, 2021 [73]	Oral	*SQLE*	Adverse prognosis	Positive correlation with rMC infiltration
6	Chu, 2022 [74]	HNSCC	*SLC2A3*	Adverse prognosis	Positive correlation with MC infiltration
7	Lin, 2022 [75]	HNSCC	*FCGBP*	Adverse prognosis	Positive correlation with rMC infiltration
8	Wang, 2023 [33]	HNSCC	*PGK1*	Adverse prognosis	Negative correlation with MC infiltration
9	Peng, 2023 [29]	HNSCC	*GAPDH*	NM	Hindering communication between pDC and MCs
High expression of genes involved in ageing					
10	Zhang, 2022 [25]	HNSCC	*CBX3*	Adverse prognosis	Negative correlation with MC infiltration
11	Li, 2023 [28]	HNSCC	*HOXB9*	Adverse prognosis	Negative correlation with MC infiltration
High expression of other genes					
12	Chen, 2022 [44]	Oral	*SFRP1*	Favorable prognosis	Positive correlation with MC infiltration
13	Li, 2023 [46]	HNSCC	*MYL1*	Adverse prognosis	Positive correlation with MC infiltration
14	Han, 2021 [43]	Oral	*MFAP4*	Favorable prognosis	Positive correlation with MC infiltration
High expression of lncRNA					
15	Liu, 2020 [41]	Oral	*FOXD2-AS1*	Adverse prognosis	Negative correlation with MC infiltration
16	Sun, 2022 [42]	Oral	*DDX59-AS1*	Adverse prognosis	Negative correlation with MC infiltration

aMCs: activated mast cells; HNSCC: head and neck squamous cell carcinoma; MCs: mast cells; NM: not mentioned; pDC: plasmacytoid dendritic cells; rMCs: resting mast cells; lncRNA: long non-coding RNA.

## Data Availability

No new data were created or analyzed in this study.

## Supplementary Materials

The following supporting information can be downloaded at: https://www.mdpi.com/article/10.3390/medicina60071173/s1, Supplementary File S1: The risk of bias assessment.
