# Supplementary figures and images for: The Complex Role of Mast Cells in Head and Neck Squamous Cell Carcinoma: A Systematic Review
Source: Medicina (Kaunas). 2024 Jul 19;60(7):1173. doi: 10.3390/medicina60071173 (PMC11279237; doi:10.3390/medicina60071173)

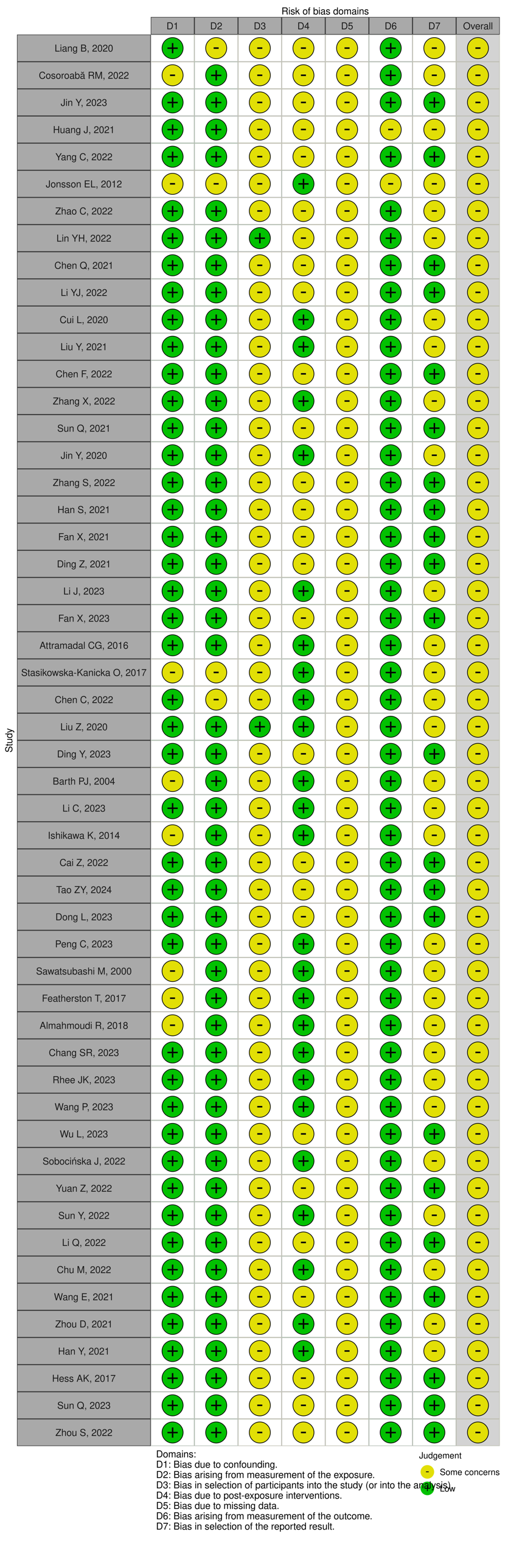

Supplement: Supplementary file 1 [file medicina-60-01173-s001.zip › medicina-3051693-supplementary.jpeg]
